# (8*S*,9*S*,10*R*)-4-(4-Chloro­benz­yloxy)-7,8-didehydro-3,7-dimeth­oxy-17-methyl­morphinan-6-one monohydrate

**DOI:** 10.1107/S1600536811013973

**Published:** 2011-04-22

**Authors:** Xing-Liang Zheng, Shu-Jun Chen, Ning-Fei Jiang, Sue-Hui Zhan

**Affiliations:** a School of Chemistry and Biological Engineering, Changsha University of Science & Technology, Changsha 410114, People’s Republic of China

## Abstract

In the title compound, C_26_H_28_Cl_N_O_4_·H_2_O, the dihedral angle betwene the two aromatic rings is 69.73 (6)°. The N-containing ring exhibits a chair conformation, while the other non-aromatic rings are in approximate envelope conformations. In the crystal, the water mol­ecule forms O—H⋯O and O—H⋯N hydrogen bonds and a C—H⋯O link also occurs.

## Related literature

For background to the biological activity of sinomenine derivatives and other related compounds, see: Liu *et al.* (1994 ▶, 1996 ▶, 1997 ▶); Mark *et al.* (2003 ▶); Ye *et al.* (2004 ▶). For the synthesis of the title compound, see: Mitsunobu (1981 ▶). For related structures, see: Li *et al.* (2009 ▶); Batterham *et al.* (1965 ▶).
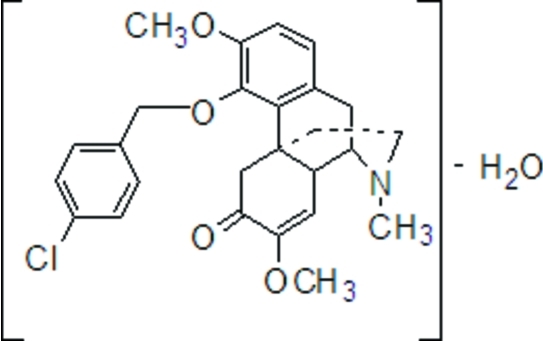


## Experimental

### 

#### Crystal data

C_26_H_28_ClNO_4_·H_2_O
*M* *_r_* = 471.96
Monoclinic, 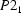

*a* = 8.8866 (4) Å
*b* = 14.6386 (7) Å
*c* = 9.1860 (4) Å
β = 91.618 (1)°
*V* = 1194.51 (9) Å^3^
*Z* = 2
Mo *K*α radiation
μ = 0.20 mm^−1^
*T* = 296 K
0.45 × 0.36 × 0.32 mm

#### Data collection

Bruker SMART CCD area-detector diffractometer
Absorption correction: multi-scan (*SADABS*; Bruker, 2000 ▶) *T*_min_ = 0.917, *T*_max_ = 0.940
13795 measured reflections
4185 independent reflections
3974 reflections with *I* > 2σ(*I*)
*R*_int_ = 0.022

#### Refinement

*R*[*F* ^2^ > 2σ(*F* ^2^)] = 0.030
*wR*(*F* ^2^) = 0.082
*S* = 1.05
4185 reflections
303 parameters
3 restraints
H atoms treated by a mixture of independent and constrained refinement
Δρ_max_ = 0.20 e Å^−3^
Δρ_min_ = −0.21 e Å^−3^
Absolute structure: Flack (1983 ▶), 1991 Friedel pairs
Flack parameter: −0.02 (6)

### 

Data collection: *SMART* (Bruker, 2000 ▶); cell refinement: *SAINT* (Bruker, 2000 ▶); data reduction: *SAINT*; program(s) used to solve structure: *SHELXS97* (Sheldrick, 2008 ▶); program(s) used to refine structure: *SHELXL97* (Sheldrick, 2008 ▶); molecular graphics: *SHELXTL* (Sheldrick, 2008 ▶); software used to prepare material for publication: *SHELXTL*.

## Supplementary Material

Crystal structure: contains datablocks global, I. DOI: 10.1107/S1600536811013973/kp2320sup1.cif

Structure factors: contains datablocks I. DOI: 10.1107/S1600536811013973/kp2320Isup2.hkl

Additional supplementary materials:  crystallographic information; 3D view; checkCIF report

## Figures and Tables

**Table 1:** Hydrogen-bond geometry (Å, °)

*D*—H⋯*A*	*D*—H	H⋯*A*	*D*⋯*A*	*D*—H⋯*A*
C8—H8*A*⋯O1^i^	0.98	2.56	3.469 (2)	155
O1*W*—H1*WB*⋯O4^ii^	0.85	2.04	2.879 (2)	169
O1*W*—H1*WA*⋯N1	0.85	2.16	2.899 (2)	146

Symmetry codes: (i) 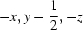; (ii) 
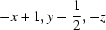
.

**Fig. 1.**
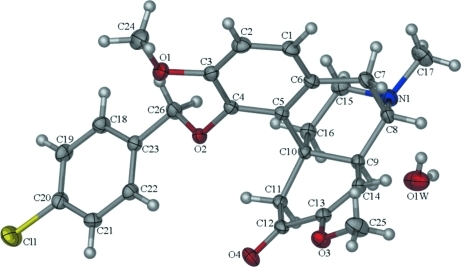
The molecular structure of (I) showing 50% probability displacement ellipsoids [Symmetry codes: (i) 1 - x, 2 - y, 2 - z].

**Fig. 2.**
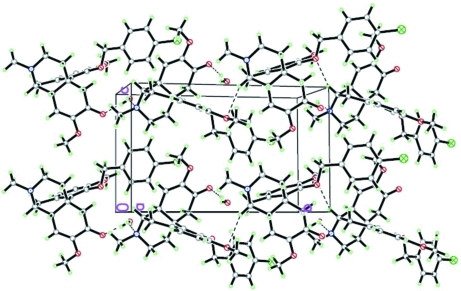
Crystal packing of (I) with hydrogen bond interactions (dashed lines).

## supplementary crystallographic information

### Comment

We synthesised a new sinomenine derivative (8*S*,9*S*,10*R*)-7,8-didehydro-4-(4'-chlorobenzyloxy)-3,7-\ dimethoxy-17-methyl-morphinan-6-one monohydrate. Herein, its crystal structure is reported. Biological effects of sinomenine derivatives and related compounds have been described (Liu *et al.*, 1994, 1996, 1997; Mark *et al.*, 2003; Ye *et al.*, 2004).

The molecular structure of (I) is shown in Fig. 1. The angle between the two benzene planes is 69.736°. Rings *C* [C9/C10/C11/C12/C13/C14] and *B* [C5···.C10], both, are in an envelope conformation. In contrast, ring *D* [C8/N1/C15/C16/C10/C9] exhibits an almost regular chair conformation. Similar features have been described in related compounds (Li *et al.*, 2009; Batterham *et al.*, 1965). The crystal structure is stabilised by O—H···O and O—H···N hydrogen bonds linking sinomenine derivative and the water molecule; weak C—H···O hydrogen bonds (Table 1 and Fig. 2) also occurred.

### Experimental

The title compound was obtained according to the method of Mitsunobu (1981). Colourless blocks of (I) were grown from an acetic ether solution.

### Refinement

The water H atoms (H5C and H5D) were located in a difference map, and refined freely, although the geometry was restrained to O—H = 0.83 (3) Å and H5C···H5D separation to 1.45 (2) Å. Other H atoms were positioned geometrically, with C—H = 0.93 (aromatic CH), 0.96 (methyl CH_3_), 0.97 (methylene CH_2_) or 0.98 Å (methine CH), and were constrained to ride on their parent atoms, with *U*_iso_(H) = 1.2*U*_eq_(carrier C) or *U*_iso_(H) = 1.5*U*_eq_(carrier C). 2311 Friedel pairs were used for the Flack parameter refinement.

### Crystal data

**Table:**

C_26_H_28_ClNO_4_·H_2_O	*F*(000) = 500
*M**_r_* = 471.96	*D*_x_ = 1.312 Mg m^−^^3^
Monoclinic, *P*2_1_	Melting point: 383 K
*a* = 8.8866 (4) Å	Mo *K*α radiation, λ = 0.71073 Å
*b* = 14.6386 (7) Å	µ = 0.20 mm^−^^1^
*c* = 9.1860 (4) Å	*T* = 296 K
β = 91.618 (1)°	Block, colourless
*V* = 1194.51 (9) Å^3^	0.45 × 0.36 × 0.32 mm
*Z* = 2	

### Data collection

**Table:**

Bruker SMART CCD area-detector diffractometer	4185 independent reflections
Radiation source: fine-focus sealed tube	3974 reflections with *I* > 2σ(*I*)
graphite	*R*_int_ = 0.022
φ and ω scans	θ_max_ = 25.0°, θ_min_ = 2.2°
Absorption correction: multi-scan (*SADABS*; Bruker, 2000)	*h* = −10→10
*T*_min_ = 0.917, *T*_max_ = 0.940	*k* = −17→17
13795 measured reflections	*l* = −10→10

### Refinement

**Table:**

Refinement on *F*^2^	Secondary atom site location: difference Fourier map
Least-squares matrix: full	Hydrogen site location: inferred from neighbouring sites
*R*[*F*^2^ > 2σ(*F*^2^)] = 0.030	H atoms treated by a mixture of independent and constrained refinement
*wR*(*F*^2^) = 0.082	*w* = 1/[σ^2^(*F*_o_^2^) + (0.0458*P*)^2^ + 0.1696*P*] where *P* = (*F*_o_^2^ + 2*F*_c_^2^)/3
*S* = 1.05	(Δ/σ)_max_ < 0.001
4185 reflections	Δρ_max_ = 0.20 e Å^−^^3^
303 parameters	Δρ_min_ = −0.21 e Å^−^^3^
3 restraints	Absolute structure: Flack (1983), 1991 Friedel pairs
Primary atom site location: structure-invariant direct methods	Flack parameter: −0.02 (6)

### Special details

**Table:**

Geometry. All e.s.d.'s (except the e.s.d. in the dihedral angle between two l.s. planes) are estimated using the full covariance matrix. The cell e.s.d.'s are taken into account individually in the estimation of e.s.d.'s in distances, angles and torsion angles; correlations between e.s.d.'s in cell parameters are only used when they are defined by crystal symmetry. An approximate (isotropic) treatment of cell e.s.d.'s is used for estimating e.s.d.'s involving l.s. planes.
Refinement. Refinement of *F*^2^ against ALL reflections. The weighted *R*-factor *wR* and goodness of fit *S* are based on *F*^2^, conventional *R*-factors *R* are based on *F*, with *F* set to zero for negative *F*^2^. The threshold expression of *F*^2^ > σ(*F*^2^) is used only for calculating *R*-factors(gt) *etc*. and is not relevant to the choice of reflections for refinement. *R*-factors based on *F*^2^ are statistically about twice as large as those based on *F*, and *R*- factors based on ALL data will be even larger.

### Fractional atomic coordinates and isotropic or equivalent isotropic displacement parameters (Å^2^)

**Table:**

	*x*	*y*	*z*	*U*_iso_*/*U*_eq_	
Cl1	0.52194 (7)	0.27734 (5)	0.40802 (7)	0.07659 (19)	
O1	−0.15952 (17)	−0.04761 (10)	0.27652 (16)	0.0553 (4)	
O2	0.12278 (13)	−0.08829 (9)	0.21247 (13)	0.0412 (3)	
O3	0.07164 (16)	−0.20241 (10)	−0.34261 (14)	0.0524 (3)	
O4	0.23766 (16)	−0.08573 (9)	−0.18937 (17)	0.0563 (4)	
N1	0.22663 (19)	−0.45473 (11)	0.17465 (19)	0.0501 (4)	
C1	−0.2087 (2)	−0.28319 (15)	0.1494 (2)	0.0487 (4)	
H1A	−0.2839	−0.3269	0.1371	0.058*	
C2	−0.2467 (2)	−0.19781 (15)	0.1984 (2)	0.0510 (5)	
H2A	−0.3467	−0.1835	0.2153	0.061*	
C3	−0.1356 (2)	−0.13375 (13)	0.22213 (19)	0.0427 (4)	
C4	0.01413 (18)	−0.15522 (11)	0.18989 (17)	0.0355 (3)	
C5	0.05174 (17)	−0.23996 (11)	0.13104 (16)	0.0337 (3)	
C6	−0.06217 (19)	−0.30630 (12)	0.11783 (19)	0.0393 (4)	
C7	−0.0312 (2)	−0.40384 (13)	0.0731 (2)	0.0473 (4)	
H7A	−0.0893	−0.4167	−0.0155	0.057*	
H7B	−0.0674	−0.4441	0.1483	0.057*	
C8	0.1333 (2)	−0.42725 (12)	0.0471 (2)	0.0441 (4)	
H8A	0.1348	−0.4775	−0.0234	0.053*	
C9	0.2069 (2)	−0.34492 (11)	−0.02251 (19)	0.0383 (4)	
H9A	0.3110	−0.3617	−0.0430	0.046*	
C10	0.21264 (18)	−0.26412 (11)	0.08633 (18)	0.0355 (3)	
C11	0.29450 (18)	−0.18780 (12)	0.00569 (19)	0.0392 (4)	
H11A	0.2997	−0.1342	0.0678	0.047*	
H11B	0.3968	−0.2074	−0.0110	0.047*	
C12	0.22199 (18)	−0.16152 (12)	−0.1371 (2)	0.0400 (4)	
C13	0.13513 (19)	−0.23343 (13)	−0.21620 (18)	0.0411 (4)	
C14	0.1311 (2)	−0.31859 (13)	−0.16395 (19)	0.0419 (4)	
H14A	0.0794	−0.3631	−0.2174	0.050*	
C15	0.2405 (2)	−0.38269 (15)	0.2849 (2)	0.0516 (5)	
H15A	0.3056	−0.4035	0.3647	0.062*	
H15B	0.1422	−0.3698	0.3234	0.062*	
C16	0.3050 (2)	−0.29596 (13)	0.2203 (2)	0.0441 (4)	
H16A	0.4080	−0.3071	0.1929	0.053*	
H16B	0.3062	−0.2481	0.2933	0.053*	
C17	0.1749 (4)	−0.54114 (17)	0.2379 (3)	0.0758 (7)	
H17A	0.2381	−0.5566	0.3208	0.114*	
H17B	0.1799	−0.5888	0.1664	0.114*	
H17C	0.0729	−0.5344	0.2679	0.114*	
C18	0.2921 (2)	0.05280 (14)	0.5062 (2)	0.0491 (5)	
H18A	0.2597	0.0239	0.5899	0.059*	
C19	0.3747 (2)	0.13208 (15)	0.5183 (2)	0.0515 (5)	
H19A	0.3979	0.1569	0.6094	0.062*	
C20	0.4226 (2)	0.17419 (14)	0.3939 (2)	0.0472 (4)	
C21	0.3920 (2)	0.13761 (14)	0.2589 (2)	0.0468 (4)	
H21A	0.4261	0.1662	0.1756	0.056*	
C22	0.3096 (2)	0.05773 (13)	0.2481 (2)	0.0433 (4)	
H22A	0.2897	0.0322	0.1569	0.052*	
C23	0.25623 (19)	0.01518 (13)	0.37027 (19)	0.0394 (4)	
C24	−0.3108 (3)	−0.02259 (19)	0.3035 (3)	0.0779 (8)	
H24A	−0.3131	0.0386	0.3410	0.117*	
H24B	−0.3517	−0.0637	0.3735	0.117*	
H24C	−0.3696	−0.0257	0.2144	0.117*	
C25	−0.0249 (3)	−0.2642 (2)	−0.4189 (2)	0.0710 (7)	
H25A	−0.0639	−0.2355	−0.5061	0.106*	
H25B	−0.1067	−0.2809	−0.3583	0.106*	
H25C	0.0306	−0.3179	−0.4440	0.106*	
C26	0.15968 (16)	−0.06997 (10)	0.36153 (16)	0.0480 (4)	
H26A	0.2140	−0.1213	0.4042	0.058*	
H26B	0.0684	−0.0609	0.4152	0.058*	
O1W	0.53363 (16)	−0.46331 (10)	0.07692 (16)	0.0895 (6)	
H1WB	0.592 (4)	−0.502 (2)	0.118 (4)	0.121 (13)*	
H1WA	0.4437 (18)	−0.481 (3)	0.084 (4)	0.116 (13)*	

### Atomic displacement parameters (Å^2^)

**Table:**

	*U*^11^	*U*^22^	*U*^33^	*U*^12^	*U*^13^	*U*^23^
Cl1	0.0763 (4)	0.0731 (4)	0.0804 (4)	−0.0350 (3)	0.0019 (3)	−0.0121 (3)
O1	0.0574 (8)	0.0452 (8)	0.0640 (9)	0.0130 (6)	0.0149 (6)	−0.0006 (6)
O2	0.0484 (7)	0.0366 (6)	0.0386 (6)	−0.0066 (5)	0.0034 (5)	−0.0041 (5)
O3	0.0583 (8)	0.0525 (8)	0.0461 (7)	−0.0059 (6)	−0.0052 (6)	0.0095 (6)
O4	0.0588 (8)	0.0376 (8)	0.0724 (9)	−0.0072 (6)	0.0007 (7)	0.0089 (7)
N1	0.0532 (9)	0.0424 (9)	0.0548 (9)	0.0022 (7)	0.0011 (7)	0.0092 (7)
C1	0.0357 (9)	0.0530 (12)	0.0576 (11)	−0.0082 (8)	0.0053 (8)	0.0031 (9)
C2	0.0348 (9)	0.0597 (13)	0.0589 (12)	0.0038 (8)	0.0082 (8)	0.0071 (9)
C3	0.0454 (9)	0.0412 (10)	0.0419 (9)	0.0072 (8)	0.0072 (8)	0.0052 (8)
C4	0.0370 (8)	0.0349 (9)	0.0345 (8)	−0.0023 (7)	0.0009 (6)	0.0031 (7)
C5	0.0327 (8)	0.0360 (9)	0.0322 (7)	−0.0021 (6)	−0.0009 (6)	0.0026 (7)
C6	0.0377 (8)	0.0406 (10)	0.0396 (8)	−0.0063 (7)	0.0013 (7)	0.0028 (7)
C7	0.0485 (10)	0.0398 (10)	0.0536 (10)	−0.0117 (8)	−0.0007 (8)	−0.0022 (8)
C8	0.0542 (10)	0.0316 (9)	0.0464 (10)	−0.0030 (8)	0.0027 (8)	−0.0013 (8)
C9	0.0405 (9)	0.0320 (9)	0.0426 (9)	0.0009 (7)	0.0059 (7)	−0.0025 (7)
C10	0.0338 (8)	0.0332 (8)	0.0396 (8)	−0.0025 (6)	0.0017 (7)	−0.0033 (7)
C11	0.0329 (8)	0.0380 (9)	0.0470 (9)	−0.0060 (7)	0.0069 (7)	−0.0063 (7)
C12	0.0339 (8)	0.0326 (9)	0.0540 (10)	−0.0007 (7)	0.0124 (7)	0.0030 (8)
C13	0.0403 (9)	0.0417 (10)	0.0415 (9)	−0.0016 (8)	0.0055 (7)	0.0003 (8)
C14	0.0483 (9)	0.0371 (9)	0.0403 (9)	−0.0048 (7)	0.0034 (7)	−0.0059 (7)
C15	0.0498 (10)	0.0588 (13)	0.0461 (10)	0.0044 (9)	−0.0010 (8)	0.0094 (9)
C16	0.0380 (8)	0.0461 (10)	0.0480 (10)	0.0018 (8)	−0.0044 (7)	−0.0040 (8)
C17	0.0948 (18)	0.0526 (14)	0.0798 (16)	−0.0033 (13)	−0.0015 (14)	0.0272 (12)
C18	0.0534 (11)	0.0556 (12)	0.0380 (9)	−0.0021 (9)	−0.0005 (8)	0.0007 (8)
C19	0.0499 (10)	0.0632 (13)	0.0410 (10)	−0.0067 (9)	−0.0045 (8)	−0.0142 (9)
C20	0.0345 (8)	0.0515 (11)	0.0556 (11)	−0.0050 (8)	0.0009 (7)	−0.0091 (9)
C21	0.0415 (9)	0.0541 (11)	0.0450 (10)	−0.0039 (8)	0.0055 (8)	0.0027 (9)
C22	0.0412 (9)	0.0494 (10)	0.0391 (9)	−0.0006 (8)	−0.0016 (7)	−0.0052 (8)
C23	0.0389 (9)	0.0396 (9)	0.0395 (9)	0.0036 (7)	−0.0008 (7)	−0.0029 (7)
C24	0.0695 (15)	0.0659 (16)	0.1001 (19)	0.0209 (13)	0.0321 (14)	−0.0023 (14)
C25	0.0798 (16)	0.0815 (17)	0.0508 (12)	−0.0221 (13)	−0.0132 (11)	0.0118 (12)
C26	0.0613 (12)	0.0406 (11)	0.0418 (9)	−0.0037 (8)	−0.0030 (8)	0.0013 (8)
O1W	0.0715 (13)	0.0886 (15)	0.1095 (15)	0.0265 (11)	0.0246 (11)	0.0396 (12)

### Geometric parameters (Å, °)

**Table:**

Cl1—C20	1.752 (2)	C11—H11B	0.9700
O1—C3	1.375 (2)	C12—C13	1.483 (3)
O1—C24	1.422 (3)	C13—C14	1.337 (3)
O2—C4	1.387 (2)	C14—H14A	0.9300
O2—C26	1.4244 (19)	C15—C16	1.521 (3)
O3—C13	1.355 (2)	C15—H15A	0.9700
O3—C25	1.418 (3)	C15—H15B	0.9700
O4—C12	1.218 (2)	C16—H16A	0.9700
N1—C15	1.465 (3)	C16—H16B	0.9700
N1—C17	1.471 (3)	C17—H17A	0.9600
N1—C8	1.473 (2)	C17—H17B	0.9600
C1—C2	1.374 (3)	C17—H17C	0.9600
C1—C6	1.384 (3)	C18—C19	1.376 (3)
C1—H1A	0.9300	C18—C23	1.393 (3)
C2—C3	1.375 (3)	C18—H18A	0.9300
C2—H2A	0.9300	C19—C20	1.377 (3)
C3—C4	1.407 (2)	C19—H19A	0.9300
C4—C5	1.397 (2)	C20—C21	1.371 (3)
C5—C6	1.406 (2)	C21—C22	1.382 (3)
C5—C10	1.540 (2)	C21—H21A	0.9300
C6—C7	1.513 (3)	C22—C23	1.379 (3)
C7—C8	1.526 (3)	C22—H22A	0.9300
C7—H7A	0.9700	C23—C26	1.514 (2)
C7—H7B	0.9700	C24—H24A	0.9600
C8—C9	1.521 (2)	C24—H24B	0.9600
C8—H8A	0.9800	C24—H24C	0.9600
C9—C14	1.497 (2)	C25—H25A	0.9600
C9—C10	1.549 (2)	C25—H25B	0.9600
C9—H9A	0.9800	C25—H25C	0.9600
C10—C16	1.532 (2)	C26—H26A	0.9700
C10—C11	1.535 (2)	C26—H26B	0.9700
C11—C12	1.495 (3)	O1W—H1WB	0.851 (10)
C11—H11A	0.9700	O1W—H1WA	0.846 (10)
			
C3—O1—C24	117.17 (18)	O3—C13—C12	112.34 (16)
C4—O2—C26	114.65 (12)	C13—C14—C9	122.41 (16)
C13—O3—C25	116.20 (16)	C13—C14—H14A	118.8
C15—N1—C17	111.53 (18)	C9—C14—H14A	118.8
C15—N1—C8	112.83 (15)	N1—C15—C16	110.88 (15)
C17—N1—C8	111.96 (18)	N1—C15—H15A	109.5
C2—C1—C6	122.20 (18)	C16—C15—H15A	109.5
C2—C1—H1A	118.9	N1—C15—H15B	109.5
C6—C1—H1A	118.9	C16—C15—H15B	109.5
C1—C2—C3	119.34 (17)	H15A—C15—H15B	108.1
C1—C2—H2A	120.3	C15—C16—C10	111.60 (14)
C3—C2—H2A	120.3	C15—C16—H16A	109.3
C2—C3—O1	124.42 (16)	C10—C16—H16A	109.3
C2—C3—C4	119.58 (17)	C15—C16—H16B	109.3
O1—C3—C4	116.00 (16)	C10—C16—H16B	109.3
O2—C4—C5	120.78 (14)	H16A—C16—H16B	108.0
O2—C4—C3	117.98 (15)	N1—C17—H17A	109.5
C5—C4—C3	121.18 (15)	N1—C17—H17B	109.5
C4—C5—C6	117.85 (15)	H17A—C17—H17B	109.5
C4—C5—C10	122.76 (14)	N1—C17—H17C	109.5
C6—C5—C10	119.35 (15)	H17A—C17—H17C	109.5
C1—C6—C5	119.51 (17)	H17B—C17—H17C	109.5
C1—C6—C7	117.85 (16)	C19—C18—C23	120.93 (18)
C5—C6—C7	122.62 (15)	C19—C18—H18A	119.5
C6—C7—C8	115.91 (15)	C23—C18—H18A	119.5
C6—C7—H7A	108.3	C18—C19—C20	119.15 (17)
C8—C7—H7A	108.3	C18—C19—H19A	120.4
C6—C7—H7B	108.3	C20—C19—H19A	120.4
C8—C7—H7B	108.3	C21—C20—C19	121.19 (18)
H7A—C7—H7B	107.4	C21—C20—Cl1	119.39 (16)
N1—C8—C9	108.14 (15)	C19—C20—Cl1	119.42 (15)
N1—C8—C7	117.17 (16)	C20—C21—C22	119.11 (18)
C9—C8—C7	108.27 (15)	C20—C21—H21A	120.4
N1—C8—H8A	107.6	C22—C21—H21A	120.4
C9—C8—H8A	107.6	C23—C22—C21	121.18 (17)
C7—C8—H8A	107.6	C23—C22—H22A	119.4
C14—C9—C8	112.35 (15)	C21—C22—H22A	119.4
C14—C9—C10	111.68 (14)	C22—C23—C18	118.39 (18)
C8—C9—C10	109.92 (14)	C22—C23—C26	122.41 (15)
C14—C9—H9A	107.6	C18—C23—C26	119.20 (16)
C8—C9—H9A	107.6	O1—C24—H24A	109.5
C10—C9—H9A	107.6	O1—C24—H24B	109.5
C16—C10—C11	111.01 (14)	H24A—C24—H24B	109.5
C16—C10—C5	109.63 (13)	O1—C24—H24C	109.5
C11—C10—C5	114.69 (14)	H24A—C24—H24C	109.5
C16—C10—C9	107.05 (14)	H24B—C24—H24C	109.5
C11—C10—C9	104.58 (13)	O3—C25—H25A	109.5
C5—C10—C9	109.50 (13)	O3—C25—H25B	109.5
C12—C11—C10	114.34 (13)	H25A—C25—H25B	109.5
C12—C11—H11A	108.7	O3—C25—H25C	109.5
C10—C11—H11A	108.7	H25A—C25—H25C	109.5
C12—C11—H11B	108.7	H25B—C25—H25C	109.5
C10—C11—H11B	108.7	O2—C26—C23	108.70 (12)
H11A—C11—H11B	107.6	O2—C26—H26A	109.9
O4—C12—C13	121.13 (17)	C23—C26—H26A	109.9
O4—C12—C11	121.91 (17)	O2—C26—H26B	109.9
C13—C12—C11	116.90 (15)	C23—C26—H26B	109.9
C14—C13—O3	127.28 (17)	H26A—C26—H26B	108.3
C14—C13—C12	120.32 (16)	H1WB—O1W—H1WA	109 (4)
			
C6—C1—C2—C3	−2.4 (3)	C8—C9—C10—C16	59.88 (18)
C1—C2—C3—O1	−177.22 (17)	C14—C9—C10—C11	−56.88 (17)
C1—C2—C3—C4	2.8 (3)	C8—C9—C10—C11	177.73 (14)
C24—O1—C3—C2	−2.9 (3)	C14—C9—C10—C5	66.49 (17)
C24—O1—C3—C4	177.09 (19)	C8—C9—C10—C5	−58.90 (18)
C26—O2—C4—C5	−110.58 (16)	C16—C10—C11—C12	172.20 (15)
C26—O2—C4—C3	72.10 (18)	C5—C10—C11—C12	−62.86 (19)
C2—C3—C4—O2	178.72 (15)	C9—C10—C11—C12	57.09 (18)
O1—C3—C4—O2	−1.3 (2)	C10—C11—C12—O4	154.14 (17)
C2—C3—C4—C5	1.4 (2)	C10—C11—C12—C13	−28.5 (2)
O1—C3—C4—C5	−178.57 (14)	C25—O3—C13—C14	8.4 (3)
O2—C4—C5—C6	176.86 (14)	C25—O3—C13—C12	−174.43 (18)
C3—C4—C5—C6	−5.9 (2)	O4—C12—C13—C14	173.55 (17)
O2—C4—C5—C10	−0.7 (2)	C11—C12—C13—C14	−3.8 (2)
C3—C4—C5—C10	176.50 (15)	O4—C12—C13—O3	−3.8 (2)
C2—C1—C6—C5	−2.2 (3)	C11—C12—C13—O3	178.80 (14)
C2—C1—C6—C7	176.47 (18)	O3—C13—C14—C9	179.57 (15)
C4—C5—C6—C1	6.2 (2)	C12—C13—C14—C9	2.6 (3)
C10—C5—C6—C1	−176.07 (16)	C8—C9—C14—C13	154.08 (17)
C4—C5—C6—C7	−172.38 (15)	C10—C9—C14—C13	30.0 (2)
C10—C5—C6—C7	5.3 (2)	C17—N1—C15—C16	175.80 (18)
C1—C6—C7—C8	−176.95 (16)	C8—N1—C15—C16	−57.2 (2)
C5—C6—C7—C8	1.7 (3)	N1—C15—C16—C10	54.9 (2)
C15—N1—C8—C9	60.6 (2)	C11—C10—C16—C15	−169.38 (15)
C17—N1—C8—C9	−172.57 (18)	C5—C10—C16—C15	62.88 (19)
C15—N1—C8—C7	−62.0 (2)	C9—C10—C16—C15	−55.82 (18)
C17—N1—C8—C7	64.8 (2)	C23—C18—C19—C20	−0.2 (3)
C6—C7—C8—N1	86.1 (2)	C18—C19—C20—C21	−1.3 (3)
C6—C7—C8—C9	−36.5 (2)	C18—C19—C20—Cl1	177.68 (16)
N1—C8—C9—C14	172.93 (14)	C19—C20—C21—C22	0.9 (3)
C7—C8—C9—C14	−59.18 (19)	Cl1—C20—C21—C22	−178.01 (15)
N1—C8—C9—C10	−62.06 (18)	C20—C21—C22—C23	0.9 (3)
C7—C8—C9—C10	65.83 (18)	C21—C22—C23—C18	−2.4 (3)
C4—C5—C10—C16	83.19 (19)	C21—C22—C23—C26	177.14 (16)
C6—C5—C10—C16	−94.37 (18)	C19—C18—C23—C22	2.0 (3)
C4—C5—C10—C11	−42.5 (2)	C19—C18—C23—C26	−177.50 (16)
C6—C5—C10—C11	139.97 (15)	C4—O2—C26—C23	−169.61 (13)
C4—C5—C10—C9	−159.65 (14)	C22—C23—C26—O2	−6.9 (2)
C6—C5—C10—C9	22.8 (2)	C18—C23—C26—O2	172.64 (16)
C14—C9—C10—C16	−174.73 (14)		

### Hydrogen-bond geometry (Å, °)

**Table:**

*D*—H···*A*	*D*—H	H···*A*	*D*···*A*	*D*—H···*A*
C8—H8A···O1^i^	0.98	2.56	3.469 (2)	155
O1W—H1WB···O4^ii^	0.85	2.04	2.879 (2)	169
O1W—H1WA···N1	0.85	2.16	2.899 (2)	146

Symmetry codes: (i) −*x*, *y*−1/2, −*z*; (ii) −*x*+1, *y*−1/2, −*z*.
